# Time-course and mechanisms of homeostatic plasticity in layers 2/3 and 5 of the barrel cortex

**DOI:** 10.1098/rstb.2016.0150

**Published:** 2017-03-05

**Authors:** Stanislaw Glazewski, Stuart Greenhill, Kevin Fox

**Affiliations:** 1 School of Life Sciences, Keele University, Keele 5T5 5BG, UK; 2 School of Life and Health Sciences, Aston University, Birmingham B4 7ET, UK; 3 School of Biosciences, Cardiff University, Cardiff CF10 3AX, UK

**Keywords:** synapse, LTP, experience-dependent, LTD, synaptic scaling, sensory cortex

## Abstract

Recent studies have shown that ocular dominance plasticity in layer 2/3 of the visual cortex exhibits a form of homeostatic plasticity that is related to synaptic scaling and depends on TNFα. In this study, we tested whether a similar form of plasticity was present in layer 2/3 of the barrel cortex and, therefore, whether the mechanism was likely to be a general property of cortical neurons. We found that whisker deprivation could induce homeostatic plasticity in layer 2/3 of barrel cortex, but not in a mouse strain lacking synaptic scaling. The time-course of homeostatic plasticity in layer 2/3 was similar to that of L5 regular spiking (RS) neurons (L5RS), but slower than that of L5 intrinsic bursting (IB) neurons (L5IB). In layer 5, the strength of evoked whisker responses and *ex vivo* miniature excitatory post-synaptic currents (mEPSCs) amplitudes showed an identical time-course for homeostatic plasticity, implying that plasticity at excitatory synapses contacting layer 5 neurons is sufficient to explain the changes in evoked responses. Spontaneous firing rate also showed homeostatic behaviour for L5IB cells, but was absent for L5RS cells over the time-course studied. Spontaneous firing rate homeostasis was found to be independent of evoked response homeostasis suggesting that the two depend on different mechanisms.

This article is part of the themed issue ‘Integrating Hebbian and homeostatic plasticity’.

## 1. Introduction

Changes in sensory experience can drive both potentiation and depression of sensory responses in the cerebral cortex. To date, studies aimed at understanding the synaptic plasticity mechanisms underlying experience-dependent potentiation (EDP) and depression in the cerebral cortex have largely examined the possibility that LTP and LTD fulfil this role [1,2]. Studies have shown that LTP and LTD mechanisms certainly do exist in the cortex. For example, in the barrel cortex the layer 4 to layer 2/3 pathway is capable of undergoing both LTP [3–5] and LTD [5,6] as are connections between layer 5 neurons [7]. Furthermore, the relationship between the two types of plasticity is extremely close; EDP and LTP depend on the same critical factors as one another, such as CaMKII [8,9], GluA1 and nitric oxide synthase [4,10]. In developing animals, LTD and experience-dependent depression (EDD) depend on cannabinoid signalling [11,12]. Further evidence comes from studies that show that experience-dependent plasticity interacts with synaptic plasticity in a way that might be predicted if one depended on the other: for example, in the barrel cortex EDD occludes LTD and enhances LTP [5,13,14].

While evidence has been found supporting a role for LTP and LTD mechanisms in experience-dependent plasticity, studies in cell culture have revealed that a third type of synaptic plasticity mechanism exists, known as synaptic scaling [15]. Synaptic scaling tends to change the synaptic weights such as to restore the cells’ initial level of excitability and, therefore, fulfils a homeostatic function [16]. In addition to the general homeostatic nature of synaptic scaling, a subclass of mechanisms known as multiplicative synaptic scaling has the further property of maintaining the relative synaptic weights for each cell while restoring overall excitability, which has the additional benefit of not disrupting coding of information during homeostasis [17].

Studies in visual cortex suggest that synaptic scaling mechanisms may exist *in vivo* too. The dependence of synaptic upscaling on TNFα [18] and the discovery of a sub-strain of mice lacking synaptic upscaling (C57BL/6OlaHsd) [19] have allowed the role of scaling in *in vivo* EDP to be evaluated. In the visual cortex, TNFα knockout mice were found to lack open eye potentiation even though LTP was intact in slices prepared from knockouts [20]. This suggests that not only is synaptic scaling required for ocular dominance plasticity in the critical period, but also that LTP is not. Separate studies on ocular dominance plasticity in a Harlan sub-strain of mice (C57BL/6OlaHsd) showed that these mice lack synaptic scaling and open eye potentiation during the critical period [19]. However, synaptic scaling was not required for ocular dominance plasticity in the adult, but CaMKII autophosphorylation was [19], suggesting that synaptic scaling is particularly important during plasticity that occurs in early development. This notion is consistent with the idea that NMDA-dependent plasticity may dominate in adult visual cortex [21].

In this study, we wanted to know how generalizable these homeostatic mechanisms were to somatosensory cortex. In particular, we wanted to investigate plasticity in layer 2/3 neurons, a layer where TNFα and synaptic scaling-dependent plasticity had been identified in the visual cortex. While a great deal of evidence implicates LTP and LTD mechanisms in layer 2/3 of the barrel cortex, it is unclear whether this is (i) because the critical period for plasticity is so much earlier in barrel cortex than visual cortex [22,23] and, therefore, synaptic scaling has waned at the ages investigated (one to two months of age), (ii) because somatosensory and visual cortex are intrinsically different from one another, or (iii) because Hebbian and homeostatic forms of plasticity coexist in barrel cortex and have yet to be identified. To test for homeostatic plasticity, we used a form of deprivation designed to induce EDD without creating synaptic competition and thereby avoided the complications of Hebbian forms of potentiation taking place at the same time. We therefore deprived all the whiskers by trimming them unilaterally and maintained the deprivation for several days to see if the responses recovered back towards baseline after the initial depression. Depriving all the whiskers is known to cause synaptic scaling in layer 5 of the barrel cortex [24]. We studied this form of plasticity in C57BL/6J mice and in C57BL/6OlaHsd mice that lack synaptic scaling [19]. Finally, in the second part of the study, we compared the results obtained for layer 2/3 cells with data obtained from homeostatic plasticity experiments in layer 5 cells to understand commonalities and differences between pyramidal cell types.

## 2. Material and methods

### (a) Animals

Extracellular recordings were made from neurons of layer 2/3 in nine undeprived (122 neurons) and 17 deprived (246 neurons) C57BL/6J strain, and five undeprived (57 neurons) and 18 deprived (191 neurons) C57BL/6OlaHsd strain mice aged four weeks at the time of vibrissae deprivation. Only neurons located in barrel columns were included in the analysis. Additionally, a smaller number of layer IV neurons were recorded across control and deprivation groups of the same animals (83 neurons from C57BL6/J and 80 from C57BL/6OlaHsd). *In vivo* intracellular recordings were made from layer 5 neurons in seven undeprived (27 cells) and 26 deprived C57BL/6 J mice (82 cells) aged 4–10 weeks. *In vitro* intracellular recordings were made from three undeprived (20 cells) and nine deprived C57BL/6 J animals (60 cells) aged four to six weeks.

### (b) Whisker deprivation

To evoke homeostatic plasticity for extracellular recording experiments, all vibrissae were trimmed unilaterally to the length of 1–2 mm for 1, 3, 7 or 14 days, re-trimmed every second day to the same length as necessary and re-attached to the stubs on the recording day with use of cynoacrylate glue (Henkel Ltd., Winsford, UK). For intracellular recording experiments, the D-row whiskers were trimmed as far back as possible while leaving a small stump for easy reattachment prior to the recording session. Before recordings, trimmed whiskers were replaced for recording by the corresponding whiskers from the contralateral side, attached with cyanoacrylate glue.

### (c) Layer 2/3 *in vivo* extracellular recordings

#### (i) Anaesthesia and surgery

For all extracellular recording experiments anaesthesia was induced with isoflurane and maintained with urethane (1.5 g per kg of body weight, Sigma) with trace amount of acepromazine (approx. 1 mg kg^−1^ or less) injected IP. The depth of anaesthesia was monitored during the experiment and kept at III-3 stage of anaesthetic level, characterized by a sluggish hindlimb pinch reflex and delta waves in the 1–2 Hz range with occasional spindles. Small supplementary injections were made if necessary with 10% of the original dose. Body temperature was monitored throughout the experiment and maintained at 37°C using a rectal thermometer connected to a heating blanket (Harvard Apparatus, Holliston, USA). For recording, the skull was thinned over the barrel cortex with the dental drill. Before each electrode penetration, a small hole that was just large enough for the electrode to enter was made in the thinned skull using a gauge 30 hypodermic needle using gauge 30 hypodermic needle.

#### (ii) Electrodes and recording

Custom-made glass-insulated carbon fibre microelectrodes were used to record from the cortex [25]. Action potentials were recorded using Neurolog system (Digitimer, Welwyn garden City, UK) and filtered between 0.7 and 7 KHz with a 50 Hz notch filter. The signals were amplified 2000 times and digitized. During recording, neurons were sampled at roughly 50 µm depth intervals. Spontaneous firing and also vibrissa deflection-driven firing were used to isolate a given cell with use of window discriminator.

The stimulus consisted of a vertical deflection of a single contralateral whisker lasting 10 ms. For every neuron, 50 stimuli were delivered at 1 Hz using a fast piezoelectric bimorph wafer attached to a lightweight glass capillary driven from a voltage source (DS-2, Digitimer, Welwyn Garden City, UK) under control of Spike2 software (CED, Cambridge, UK). The single whisker stimulator was moved sequentially between whiskers within the receptive field. Evoked spikes were counted from 3 to 53 ms post-stimulus and the spontaneous activity rate subtracted.

#### (iii) Histological identification

For the extracellular recording experiments, at the end of each electrode penetration a small lesion was made in layer IV (1 µA, DC, 10 s, tip negative). This served to mark the location of each penetration. After each experiment, the animal was deeply anaesthetized and perfused through the heart initially with 0.1 M phosphate-buffered saline, which was followed by 4.0% buffered solution of formaldehyde. The brain was removed, the cortex flattened as described before [26] and left overnight, but no longer, in 30% sucrose in buffered solution of formaldehyde before transferring to buffered solution of just 30% sucrose. Sections were cut at 40 µm tangentially to the surface of flattened cortex using freezing microtome and the tissue was reacted for cytochrome oxidase [27]. Stained sections were later analysed under the microscope with use of the camera lucida to identify the location of lesions relative to the barrel map and to correct the recording depths where necessary.

### (d) Layer 5 *in vivo* intracellular recordings

#### (i) Anaesthesia and surgery

Anaesthesia was induced with isoflurane and maintained with urethane (1.0 g kg^−1^, with a trace amount of acepromazine of approx. 1 mg kg^−1^ or less, IP injection). Throughout the experiment a consistent depth of anaesthesia was maintained via breathing rate monitoring and observation of hind-paw reflexes. If necessary supplementary doses of urethane (0.1 g kg^−1^) were administered during the recording session. The D-row was located prior to surgery with intrinsic signal imaging using 700 nm light, an Optical Imaging 3001 ISI system and custom matlab code. A single whisker was deflected at 5 Hz every 8 s using a piezoelectric wafer. The D1, D2 and D3 barrels were identified and located relative to the surface blood vessel pattern.

After functional imaging, a small craniotomy was performed above the identified location of the D2 barrel. The final layer of bone and the dura mater were removed with a small-bore hypodermic needle. To place the carbon fibre ground electrode, a similar craniotomy was made in the posterior parietal cranium.

#### (ii) Intracellular electrodes and recordings

Borosilicate glass sharp pipettes (50–120 MΩ) were passed through the resected dura into the D2 barrel and the craniotomy was then covered with agar for stability. Recordings were performed in bridge mode with an Axoclamp 2B (Molecular Devices, CA, USA), using manual bridge balance and capacitance compensation. Data were acquired and experiments controlled through a CED Micro-1401 digitizer (CED) and Spike2 software (CED). After penetration, layer 5 cells were identified as regular spiking (RS) or intrinsic bursting (IB) based on their pattern of spiking in response to injected depolarizing current.

Whiskers were stimulated using a custom-made 3 × 3 piezoelectric actuator matrix [28] controlled by a CED3901 stimulator unit. Receptive fields were mapped with sparse noise delivered at 5 Hz in blocks of 10 (one deflection of each whisker plus a background rate recording per block) interleaving stimuli for each whisker in a pseudo-random sequence. Background firing was calculated by taking a 50 ms sample from each blank stimulus field throughout the recording (3–53 ms), the same time period as would be analysed for spikes after a normal stimulus event. Data were analysed and extracted using custom CED Spike2 and R scripts.

### (e) *In vitro* mEPSC measurements

Mice were killed by cervical dislocation, decapitated, and their brains rapidly removed and cooled in ice-cold choline dissection buffer (in mM: 108 choline-Cl, 3 KCl, 26 NaHCO_3_, 1.25 NaH_2_PO_4_, 25 d-glucose, 3 Na-pyruvate, 1 CaCl_2_, 6 MgSO_4_, 285 mOsm, bubbled with 95% O_2_ 5% CO_2_). Tangential slices (350 µm) angled across the barrel rows of the S1 region at 50° to the midline [29] and contralateral to the deprived whiskers were cut on a Microm HM650 V vibrating microtome, before being transferred to a custom-built holding chamber filled with normal artificial cerebrospinal fluid (in mM: 119 NaCl, 3.5 KCl, 1 NaH_2_PO_4_, 10 d-glucose, 2 CaCl_2_, 1 MgSO_4_, 300 mOsm bubbled with 95% O_2_ 5% CO_2_). Slices were incubated after cutting for 45 min at 32°C then returned to room temperature for 30 min before recording. Barrels were located under brightfield illumination and cells located using differential interference contrast on an Olympus BX50WI microscope. The D-row barrel was identified by counting across the five barrel rows (E medial, A most lateral). RS and IB cells were recorded at random throughout layers Va and Vb using borosilicate glass patch electrodes (4–8 MΩ) containing a potassium gluconate internal solution (in mM: 110 K-gluconate, 10 KCl, 2 MgCl_2_, 2 Na_2_ATP, 0.03 Na_2_GTP, 10 HEPES, 0.5% Biocytin, pH 7.3, 270 mOsm). 1 µM tetrodotoxin, 10 µM picrotoxin and 50 µM AP-V were added to the perfusate after identification of cell type through spiking response. Recordings were made with an Axon Multiclamp 700B amplifier, acquired and controlled with a CED Micro1401 and CED Signal software, and mEPSCs analysed using Axograph software.

### (f) Statistics

For the layer 2/3 *in vivo* recordings, one- or two-way ANOVA statistics were run followed by post hoc *t*-tests where effects were evident. Responses of neurons to whisker stimulation were averaged within each animal and animal averages compared across treatment groups. The numbers of layer 4 neurons were too few per animal to consider averaging within animals and were averaged across age cohorts.

For the layer 5 *in vitro* mEPSC recordings, data were acquired with CED Signal software and analysed with Axograph software. A random sample of 100 contiguous events were taken from each cell and combined to make one average dataset for each cohort. Cumulative probability distribution functions were generated and Kolmogorov–Smirnov (KS) tests performed using GraphPad Prism 6. Scaling was assessed by comparing the ratio of cohort means, multiplying one dataset by this ratio and comparing fits with the target cumulative distribution function using a KS test.

For the layer 5 *in vivo* intracellular recordings, spike data were extracted using custom CED Spike2 scripts and analysed with GraphPad Prism 6. Data were analysed across each time cohort with one- and two-way ANOVA and Tukey's post hoc tests as required.

## 3. Results

### (a) Homeostatic plasticity in cortical layer 2/3 neurons

**Figure 1.**
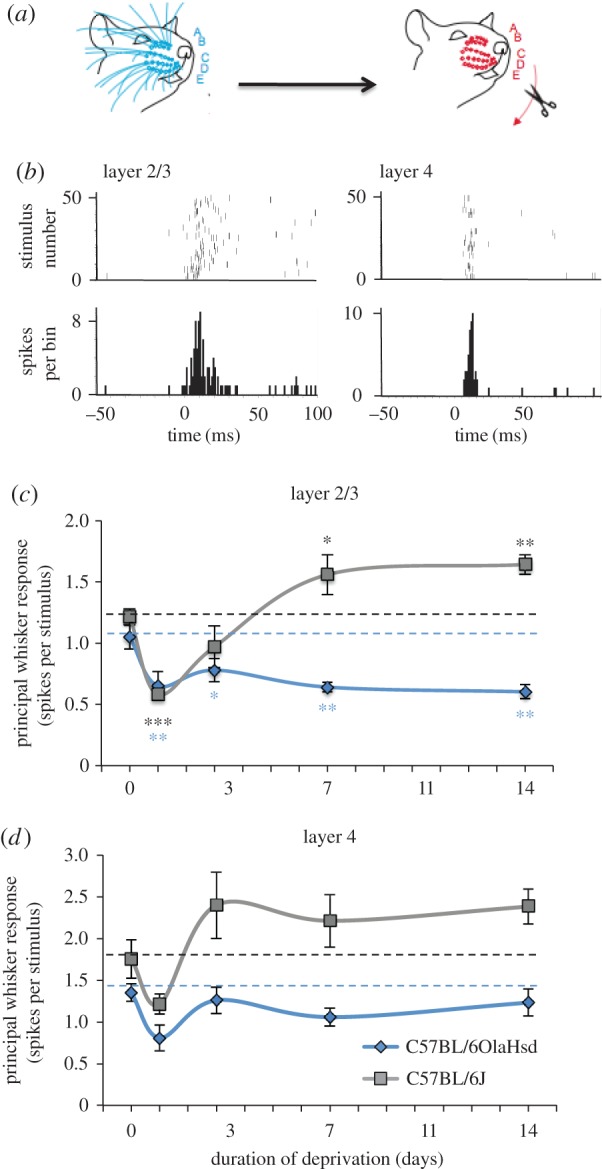
Evidence for homeostatic plasticity in layers 2/3 of barrel cortex. (*a*) All the whiskers on one side were deprived by trimming for a period of 1, 3, 7 or 14 days before recording from neurons in barrel cortex. A,B,C,D,E denote the whisker rows. (*b*) Examples of neuronal responses to principal whisker stimulation in raster (top) and post stimulus time histogram (bottom) format, generated from extracellular recordings from layer 2/3 and layer 4 (1 ms bin width, 50 stimuli). (*c*) In C57BL/6 J mice (grey line and square symbols), whisker trimming caused depression of the average layer 2/3 neuronal responses to principal whisker stimulation after 1 day (ANOVA followed by post hoc *t*-test, *t*_13_ = 7.29, *p* < 0.001, *n* = 15 mice). After 3 days some recovery occurred (not different from baseline, *t*_12_ = 1.63, *p* = 0.13, *n* = 14 mice) and by 7 days the responses were above baseline (*t*_11_ = 2.67 *p* < 0.05, *n* = 13 mice) and maintained at 14 days (*t*_10_ = 3.51, *p* < 0.01, *n* = 12 mice). In C57BL/6OlaHsd mice, depression also occurred after 1 day (*t*_12_ = 2.53, *p* < 0.05, *n* = 14 mice) but this was not followed by recovery towards baseline at any time-point (*t*_23_ = 2.06, *p* < 0.05, *n* = 25 mice). (*d*) In layer 4, neurons showed similar tendencies as in layer 2/3, however, none of the changes reached statistical significance (*p* ≥ 0.05). Data points depict means and standard errors. Dashed lines represent baseline values before deprivation. (For differences between each time-point and baseline, ***p < 0.001, **p < 0.01, *p < 0.05.)

We deprived all the whiskers unilaterally by trimming them for a period of 1, 3, 7 or 14 days (figure 1*a*) and then measured the response to a standard whisker stimulus having reattached intact whiskers to the stubs of the trimmed whiskers (figure 1*b*, see §2). Neurons were sampled evenly every 50 µm throughout the depth of layer 2/3 and 4. We recorded responses in both septal columns and barrel columns, which were identified from the location of micro-lesions made in layer 4 at the end of each recording penetration in post-mortem histology. The principal whisker is defined as the whisker that corresponds topologically to the barrel-column in which the recording is made. This determination was often ambiguous for septal locations and so we only consider penetrations made in barrel columns for the purposes of the analysis in this study.

For the control C57BL/6 J (Jackson strain) mice, we found that principal whisker responses depressed rapidly after just 1 day (24 h) of deprivation to 48% of baseline values (figure 1*c*). However, after 3 days, some recovery was found. On average, principal whisker responses recovered to 80% of control values after 3 days deprivation. We noted greater variability from animal to animal at 3 days compared with the other time-points and one animal had recovered completely (to 130% of the control mean) and another not at all (48% of control mean). This could indicate that the exact rate of recovery varies slightly from animal to animal. Later than 3 days, recovery was more uniform and, on average, layer 2/3 neuronal responses appeared to overshoot the control value at 127% by 7 days and 135% at 14 days. The depression seen after 1 day of deprivation was highly statistically significant, as was the overshoot in recovery at 7 and 14 days (figure 1*c*).

To test whether a similar homeostatic recovery was evident in animals lacking synaptic scaling, we performed the same time series of deprivations in C57BL/6OlaHsd mice. These mice have been shown to lack synaptic scaling in the visual cortex [19]. The principal whisker responses of layer 2/3 neurons showed depression after 24 h to 62% of control values and a slight, but insignificant, recovery at 3 days (74%). Furthermore, later than 3 days the responses decreased without any sign of a homeostatic recovery either at 7 days (61%) or beyond (57%) (figure 1*c*).

Neurons recorded in layer 4 appeared to show parallel changes in principal whisker response over the same deprivation period (figure 1*d*). However, none of the apparent changes seen in layer 4 neurons were statistically significantly different from baseline either for C57Bl/6 J or C57BL/6OlaHsd mice (*α* = 0.05). Nevertheless, the correlation between layer 2/3 and layer 4 principal whisker responses was significant within each animal for the C57Bl/6 J mice (*R*^2^ = 0.38, *p* < 0.005, *t*-test). To analyse the possible effect of layer 4 responses on layer 2/3, we calculated the ratio of average principal whisker responses between layer 2/3 and layer 4 neurons. This value is relatively consistent between animals and is even relatively stable with changes in anaesthesia [30]. The baseline ratio was 0.70 for C57BL/6 J mice and 0.78 for C57BL/6OlaHsd.

**Figure 2.**
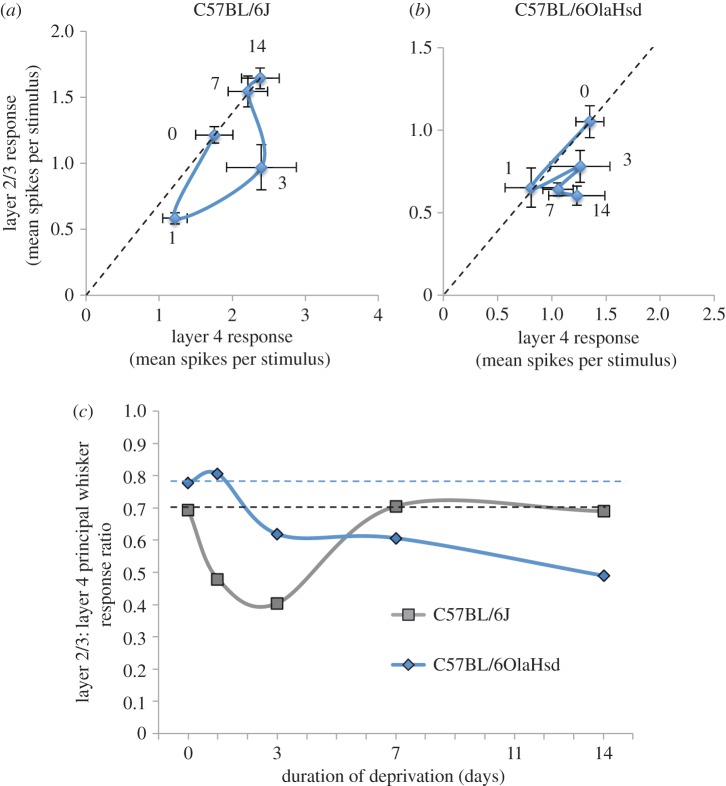
The layer 2/3 component of homeostatic plasticity. (*a*) The time-course of the change in mean principal whisker response is plotted for layer 2/3 neurons versus layer 4 neurons recorded in the same animals for C57Bl/6 J mice. The depression is almost identical in both layers after one day and the recovery in layer 2/3 is slower than in layer 4. (*b*) The depression in layers 2/3 and 4 at 1 days is almost identical (note dashed line), but beyond that time-point there is little recovery or change in the layer 2/3 response amplitude. (*c*) The ratio between the layer 2/3 and the layer 4 response are plotted for both sub-strains of mice. The C57BL/6 J mice (grey line and square symbols) show a recovery to the original baseline value whereas the C57BL/6OlaHsd mice (blue line and diamond symbols) gradually drift to lower values. Dashed lines depict original ratio of layer 2/3 : 4 before deprivation. Data points in (*a*) and (*b*) show means and standard errors. (Online version in colour.)

For C57Bl/6 J mice, when compensation for layer 4 changes is applied, the layer 2/3 component of depression demonstrates a slightly slower time-course than the uncompensated rate of depression (figure 2). The layer 2/3 component of the principal whisker response was still depressed at 3 days (ratio = 0.40) and returned to baseline by 7 days (0.70) and beyond (0.68). This shows that the rapid component of recovery seen in the overall response was largely owing to the dynamics of the layer 4 homeostatic response (figure 2). It also suggests that the overshoot seen in the layer 2/3 response is owing to an increase in layer 4 transmission rather than a gain change in the layer 4 to layer 2/3 pathway. By contrast, the layer 2/3 component of the depression in C57BL/6OlaHsd mice showed a delayed onset and first became depressed at 3 days (ratio = 0.6) and did not recover thereafter, eventually dropping to 0.49 at 14 days (figure 2*c*). These results, therefore, provide evidence that synaptic scaling plays a role in homeostatic recovery from depression in layer 2/3 neurons in barrel cortex.

### (b) Homeostatic plasticity in cortical layer 5 neurons

Studies on homeostatic plasticity in layer 5 have shown that RS and IB pyramidal cells undergo TNFα-dependent homeostatic plasticity [24]. These output layer cells of the cortex have a number of influences on their responses as they are deeply embedded in the columnar circuit, receiving inputs from all the other layers and the thalamus. As a first step towards disentangling the circuit and synaptic gain components of the homeostatic response of layer 5 neurons and in order to compare our findings with those in layer 2/3 (*vide supra*), we extended our previous study of scaling in excitatory mEPSCs *ex vivo* with further deprivation time-points to see to what extent mEPSC amplitude correlated in time with the changes in principal whisker response *in vivo*.

**Figure 3.**
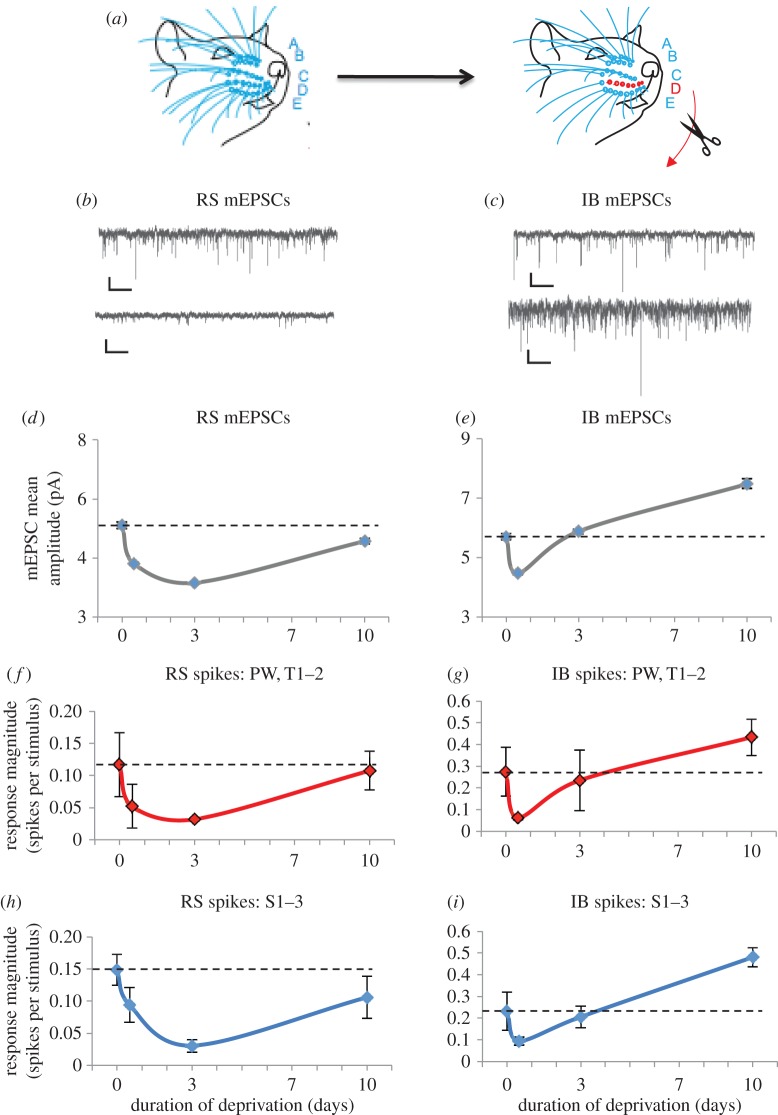
Origins of homeostatic plasticity in layer 5: correlation between the time-course of mEPSCs and whisker responses. (*a*) A single row of whiskers is deprived and recordings made in the barrels corresponding to the deprived row. Left: A,B,C,D,E denote the whisker rows. Right: deprived row D is shown in red and flanking spared rows C and E in blue. (*b*) Example miniature EPSCs recorded from a L5RS cell from an undeprived mouse (top) and a 3 day deprived mouse (bottom). (*c*) mEPSCs recorded from an undeprived mouse (top) and a 10 day deprived mouse (bottom). Scale bars 10 pA and 500 ms. (*d*) L5RS neurons' average mEPSC amplitudes decrease after 12 h of deprivation and then slowly recover towards baseline by 10 days of deprivation (control 5.2 ± 0.11 pA, 12 h 3.8 ± 0.06 pA, 10 days 4.6 ± 3.1 pA. Control versus 12 h *D* = 0.51, *p* < 0.01, KS test, 1000 events from 10 cells per group). (*e*) IB neurons show a faster recovery towards baseline by 3 days. At 10 days, mEPSC amplitudes are above baseline values (control 5.7 ± 0.11 pA, 10 days 7.5 ± 0.16 pA, *D* = 0.21, *p* < 0.01, KS test, 1000 events from 10 cells per group). (*f*) The average response of the deprived row whiskers (principal whisker and adjacent within-row whiskers) recorded from RS cells *in vivo* show a very similar time-course to the mEPSCs (*b*), only recovering after 10 days (control 0.12 ± 0.05 spikes/stim (s/s), *n* = 13 cells, 3 days 0.03 ± 0.002 s/s, *n* = 9 10 days 0.11 ± 0.03 s/s, *n* = 9). (*g*) The recovery of deprived row whiskers is faster in IB cells (control 0.27 ± 0.11 s/s, *n* = 14 cells, 12 h 0.06 ± 0.01 s/s, *n* = 14, 3 days 0.24 ± 0.14 s/s, *n* = 10) similar to the mEPSC time plot (*d*). (*h*) The spared surround whiskers with the largest responses are averaged and plotted for each time-point. The changes in response amplitude are very similar to the deprived whiskers. (*i*) The surround spared whiskers in the IB cells show a faster homeostatic recovery and potentiate beyond baseline by 10 days, similar to the mEPSCs (*c*) (control 0.23 ± 0.08 s/s, *n* = 14 cells, 10 days 0.48 ± 0.04 s/s, *n* = 9 cells, *q*_137_ = 4.55, *p* < 0.01, ANOVA with Tukey's post-hoc). Red symbols and lines show deprived whisker responses (*f* and *g*) and blue symbols and lines show spare whisker responses (*i* and *h*). Data points depict means and standard errors. Data at 0, 3 and 10 days mEPSC time-points and *in vivo* data were taken from [24].

We studied homeostasis in a row-deprivation paradigm in this case (figure 3*a*) to isolate the layer 5 changes from possible circuit effects. With the row-deprivation method, layer 4 shows a slight potentiation after 3 days and layer 2/3 does not show any change at 3 days [31]. This contrasts with layer 5 neurons, which show depression after 12 h in the case of IB neurons and 3 days in the case of RS neurons [24]. Therefore, unlike layer 2/3 cells, where a component of the apparent depression is a passive reflection of the input from layer 4 (*vide supra*), none of the major sources of cortical input to layer 5 are depressed during row-deprivation [31] even though layer 5 cells show depression at this time-point.

In RS cells, we found that mean mEPSC amplitudes (figure 3*b*) were depressed after 12 h of deprivation (mean EPSC amplitudes = 5.1 pA control, 3.8 pA 12 h, 25% depression) and continued to depress further by 3 days (3.15 pA, 17% depression, 1000 events from 10 cells per group, figure 3*d*). After 10 days of continued deprivation, mEPSCs did show some recovery and recovered to within 10% of baseline values. Several of the transitions in mEPSC amplitude between time-points exhibited multiplicative scaling. Both downscaling periods between 0 and 12 h and between 12 h and 3 days were multiplicative (0–12 h = 0.74, 12 h–3 d = 0.83). However, the upscaling period between 3 and 10 days was not multiplicative (figure 3*d*).

For IB cells, the mEPSCs were depressed after 12 h (79% of baseline) but recovered to baseline far more rapidly than was the case for the RS cells (figure 3*c,e*). After 3 days of deprivation, responses were indistinguishable from control values (103% of baseline). Beyond the homeostatic response, mEPSC amplitudes continued to potentiate, reaching 131% of baseline after 10 days (figure 3*e*). In contrast with the RS cells, the IB cells did not show multiplicative downscaling between 0 and 12 h. However, the recovery between 12 h and 3 days was multiplicative (12 h to 3 days = 1.31). The potentiation period between 3 and 10 days (figure 3*d*) was not multiplicative however, suggesting that a different process operates during homeostatic recovery compared with potentiation away from the initial set point.

The time-course of the changes in average mEPSC amplitude very closely mimic the changes in principal whisker response seen in the *in vivo* experiments [24] both for RS and IB cells, especially when reanalysing the firing rates by category of input (figure 3*f–i*), suggesting that cell-specific changes in synaptic weights are sufficient to explain the changes in sensory response without the need to invoke the participation of other neuronal circuit elements.

### (c) Plasticity of spontaneous firing rate in cortical layer 5 neurons

**Figure 4.**
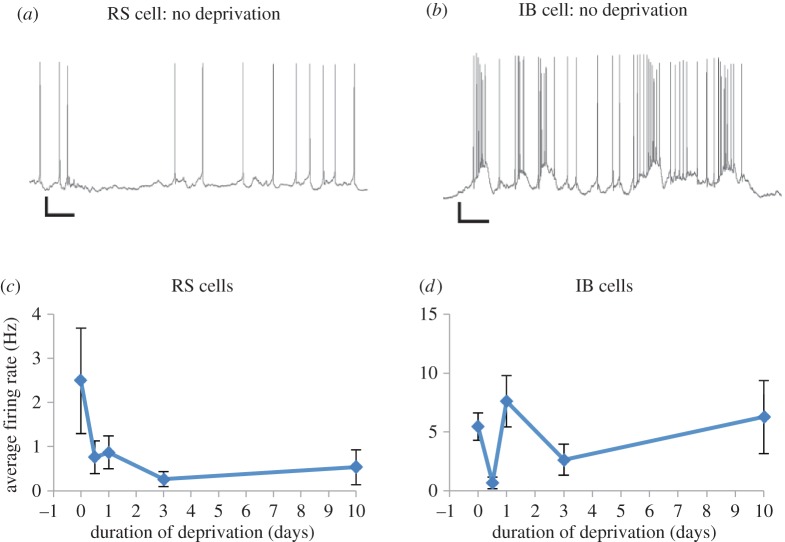
Firing rate homeostasis in IB but not RS cells. (*a*) An example of spontaneous activity recorded intracellularly *in vivo* from an RS cell in an undeprived mouse. (*b*) Spontaneous activity recorded from a L5IB cell in an undeprived mouse. Scale bars: 10 mV and 200 ms. (*c*) The spontaneous firing rate of RS cells decreases following row deprivation and does not recover by 10 days of deprivation even though the sensory responses have done so (figure 3). (*d*) The spontaneous firing rate does recover to control values in IB cells, however (control 5.46 ± 1.2 Hz, *n* = 14 cells, 10 days 6.27 ± 3.1 Hz, *n* = 9 cells, n.s., *q*_95_ = 0.63, *p* > 0.05, ANOVA with Tukey's post-hoc). Points depict means and standard errors. (Online version in colour.)

Spontaneous firing rate plasticity may or may not reflect the aggregate consequences of changes in firing rates to those circuit elements projecting to the neuron in question, together with the neuron's synaptic weights for those inputs and their intrinsic properties. Assuming for the moment that the same inputs are involved in driving spontaneous activity as are involved in producing sensory responses, then the synaptic weights determining evoked responses will be proportional to those determining spontaneous activity. If this were the case, we would expect the time-course of firing rate changes following whisker deprivation to mirror those of sensory activity. We therefore measured spontaneous activity (figure 4*a*,*b*) by taking the aggregate background activity from ‘blank’ periods of non-stimulus randomly interleaved between periods of stimulation.

We found that the assumption that spontaneous firing tracks evoked activity was approximately correct for IB cells, which showed an initial depression of spontaneous activity after 12 h followed by a jump back towards baseline values at 24 h, less recovery at 3 days and full recovery after 10 days (figure 4*d*). However, RS cells showed a depression in spontaneous activity that showed no recovery at any time-point out to 10 days (figure 4*c*). This could imply that a mechanism other than synaptic scaling of excitatory inputs produces a low level of spontaneous activity in RS cells, possibly by altering excitation coupling through intrinsic firing mechanisms [32] or by altering local somatic inhibition [33]. Alternatively, it may be that different circuit elements drive spontaneous and evoked activity in RS cells in contrast with IB cells.

## 4. Discussion

### (a) Cortical circuit versus cell-autonomous effects

Layer 2/3 and layer 5 neurons are embedded within a cortical microcircuit; any changes observed in their sensory responses might, therefore, originate from changes in other neurons within the circuit, from changes in synaptic gain of the cells in question, or a mixture of the two. In this study, we have used different methods to distinguish between these possibilities for layer 2/3 and layer 5 cells. For layer 2/3 cells we have normalized the layer 2/3 responses to the layer 4 responses to compensate for the layer 2/3 cells being strongly dominated by their columnar layer 4 input. For layer 5 cells, we have measured mEPSCs amplitudes, which report the synaptic weight of the connections on the cells in question in the absence of circuit effects (which are eliminated by TTX). Using these very different methods, we have uncovered a striking similarity in the time-course of the homeostatic rebound in layer 2/3 and in L5RS neurons (cf. figures 2*c* and 3*f*,*h*). In both cases, whisker deprivation causes a decrease in the response, reaching a minimum after approximately 3 days of deprivation followed by a homeostatic rebound back towards baseline values. By contrast, layer 5IB cells showed a faster homeostatic change than layer 2/3 or L5RS cells, and furthermore, they show input-dependent potentiation suggesting that different mechanisms operate in IB cells.

In layer 5, we found that changes in mEPSC amplitudes were strikingly similar to changes in whisker responses for both RS and IB cells. There were two main similarities: (i) both mEPSC amplitudes and sensory-evoked responses showed faster recovery in IB than RS cells and (ii) mEPSC amplitudes and sensory-evoked responses showed potentiation beyond baseline only in IB cells, whereas RS cells tend towards the original baseline values and no higher (figure 3). The close relationship between mEPSC amplitudes and sensory responses implies that, of the three most plausible candidate mechanisms for homeostatic plasticity [34]—i.e. changes in inhibition [33], changes in intrinsic membrane properties [32,35] and changes in excitatory synaptic weights [15]—changes in excitatory synaptic weights are sufficient to explain the changes in depression and recovery of sensory-evoked responses the two classes of layer 5 pyramidal cell.

The different time-course of the homeostatic response in L5RS and L5IB cells suggests that different synaptic mechanisms operate in the two cell types. While the mEPSC data suggest that only excitatory mechanisms need be considered, a number of different factors could explain the findings. Anatomical and electrophysiological studies suggest that the excitatory connections originate from different sub-circuits in the cortex [36], and RS and IB cells receive different levels of thalamic input [37]. In addition, it has been found that intrinsic plasticity mechanisms differ between RS and IB cells. While both cell types show some aspect of TNFα-dependence in their homeostatic response, only L5IB cells show a CaMKII autophosphorylation-sensitive component of plasticity [24]. One possibility is that the faster recovery rate in the IB cells is owing to the dual action of a Hebbian LTP-like synaptic plasticity mechanism operating in combination with TNFα-dependent synaptic scaling. No such mechanism operates during homeostasis in the RS cells, which could explain the slower kinetics of their recovery [24]. One further possibility is that the very property that characterizes the IB cells, namely their ability to fire a high frequency burst of action potentials, may facilitate the transmission of retrograde action potentials [38] and thereby trigger spike timing-dependent plasticity more frequently in IB than RS cells [39]. The three mechanisms mentioned here, namely divergent synaptic inputs, synaptic plasticity mechanism and intrinsic firing properties, are not mutually exclusive and may all contribute to the schism we observe between the plasticity in RS and IB cells.

### (b) Generalization of results between cortical areas

Our findings on layer 2/3 and L5RS cells generalize findings in visual cortex [19,20] and, therefore, suggest a common cortical mechanism for homeostatic plasticity. Three aspects of homeostatic plasticity are similar between the two cortical areas. First, the time-course of the layer 2/3 and L5RS cells' depression and homeostatic rebound resembles the time-course of depression and recovery observed in the visual cortex in response to monocular deprivation [20]. Second, in the case of layer 2/3 cells, synaptic scaling is likely to be a common factor between visual and somatosensory cortex. Harlan (C57BL/6OlaHsd) mice lack synaptic scaling and homeostatic response to monocular deprivation in the visual cortex [19] and lack a homeostatic response to complete whisker deprivation in the barrel cortex (figure 1). Third, synaptic scaling also requires TNFα [18] and is known to be a common factor, as no rebound from depression occurs in TNFα knockouts in layer 2/3 of visual [20] or layer 5 of somatosensory cortex [24].

It could be argued from a theoretical view point that the homeostasis seen following deprivation could be owing to a sliding threshold for LTP/LTD along the lines suggested by the Bienenstock-Cooper-Monro (BCM) theory [40]. However, it has been shown that the L5RS homeostatic response cannot be owing to classical Hebbian mechanisms, because a homeostatic rebound still occurs in the CaMKII-t286a point mutants [24], which lack LTP in hippocampus [41] and cortex [3] and lack potentiation of spared whisker responses [9].

One difference between experience-dependent plasticity in the visual and somatosensory cortex concerns the timing of critical periods. While the visual cortex is susceptible to ocular dominance plasticity especially in the final stages of development across cortical layers [23,42], in the barrel cortex the layer 4 critical period for single whisker experience ends after the first postnatal week; no critical period is seen in layer 2/3 for whisker-evoked potentiation [22], with depression in layer 2/3 present at two but not six months of life [9]. In the visual cortex, the critical period for synaptic scaling appears later in layer 2/3 than in layer 4 [43] and the critical period for ocular dominance plasticity is later in layer 2/3 and 5 than 4 [44]. However, the exact timings are shifted considerably for the two cortical areas partly because excitatory transmission between layer 4 and layer 2/3 develops at least two weeks later in the visual cortex of mice than in the somatosensory cortex [45]. The homeostatic plasticity seen in mouse visual cortex at P23-33 is, therefore, observed at a far earlier stage of development than the homeostatic plasticity in the somatosensory cortex observed in this study at one to two months of age (P28–P42). Despite this difference in developmental timings, it would appear that similar homeostatic mechanisms operate in the two areas.

In addition to the presence of homeostatic upscaling in both visual and somatosensory cortex, there is evidence that an LTD-type process is also present in both areas at the ages studied. This is perhaps not entirely surprising because without a rapid depression mechanism there would be no deviation from baseline, which might be the trigger for homeostatic potentiation. In the barrel cortex, LTD has a critical period in layer 2/3 ending around P50 in the mouse [46] and the animals described in this study were deprived of whiskers and underwent depression of whisker responses within this period. In the visual cortex, LTD shows developmental downregulation [47] and heightened sensitivity during the critical period for ocular dominance plasticity, which is thought to be owing to a peak in mGluR5 expression, as this receptor mechanism potentiates NMDA-dependent LTD [48].

Evidence that EDD in the barrel cortex is mechanistically similar to LTD comes from studies showing that both depend on the GluR1 subunit of the AMPA receptor in the somatosensory cortex [30] and the fact that LTD can be occluded by whisker deprivation patterns that cause EDD [5,6]. Crucially, EDD requires cortical activity [49], consistent with an anti-correlation mechanism of depression in barrel cortex [50]. Evidence that an LTD-like process operates during visual cortical depression of the closed eye response comes from studies showing that blocking AMPA receptor internalization prevents LTD and ocular dominance plasticity [51] and that LTD can be occluded by monocular deprivation [52].

In conclusion, both depression and homeostatic upscaling mechanisms appear to be similar between visual and somatosensory cortex and it remains to be determined whether this is also the case for non-sensory cortical association areas.

### (c) Sufficiency of the timescale of homeostasis

Modelling studies have emphasized the importance of homeostatic mechanisms for preventing the runaway effects of Hebbian synaptic processes [53–55]. The response time of homeostasis is particularly important in this regard and it has been suggested that homeostatic mechanisms need to be as fast as Hebbian mechanisms (seconds or minutes) in order to control runaway strengthening of synapses [56] that could lead to saturation of the circuit and possibly excitotoxic or epileptic effects. In this study we looked at upscaling homeostasis, which appears to be far slower than the proposed timescale of minutes. One resolution of this apparent paradox may be that we are studying upscaling and not downscaling. Downscaling is the appropriate mechanism that would be necessary to prevent runaway potentiation. A candidate for controlling potentiation, at least in the short term, may be inhibition. If feedback inhibition scales with the increased excitation produced by Hebbian potentiation, it could control the response of the cell over the short term, while a slower downscaling process mediates the longer term homeostatic response. Regarding the relatively slow kinetics of upscaling seen in this study, slow upscaling may be a safer system than a fast upscaling process for the very same reasons as a control of Hebbian runaway potentiation has been proposed: a fast upscaling process might need to be controlled so as not to saturate or cause excitotoxic effects. Even the fastest homeostatic response we observed in the layer 5IB cells takes days to return the response to baseline.

### (d) Firing rate homeostasis

A further difference between L5RS and L5IB cells was found in their firing rate homeostasis. While IB cells showed a homeostatic restoration of their basal firing rates, the RS cells showed an uncompensated loss of firing rate despite a rebound homeostasis of their evoked responses (figures 3 and 4). This result implies that spontaneous firing rate homeostasis does not necessarily depend on the synaptic weights of the excitatory inputs. Other possible mechanisms that could account for changes in firing rate include changes in inhibition [57] and changes in spike threshold or intrinsic membrane properties, for which there is some evidence in layer 5 cells [58,59]. One further possibility is that the spontaneous activity of the layer 5 neurons may be under the influence of a subset of synapses that do change synaptic weight, but cannot be detected (using mEPSC analysis) within the greater pool of synapses related to the sensory responses, which change in a different direction. Spontaneous activity of layer 5 cells is dominated by up and down states in anaesthetized animals and leads to a burst pause firing of action potentials [60]. There is evidence that the spontaneous activity of layer 5 cells depends on the intralaminar nucleus of thalamus acting via NMDA receptors [61] and this input is independent of the sensory thalamic input from the ventrobasal thalamus. It is not clear at present why such a mechanism would differ between L5RS cells compared with L5IB cells. However, it does give RS cells an adaptive advantage because the signal to noise ratio increases for L5RS cells [62] through a homeostatic response to sensory inputs and a lack of firing rate homeostasis. In this way, the L5RS cells achieve a similar result to the L5IB cells that do show firing rate homeostasis, but IB cells require a CaMKII-dependent mechanism to potentiate their spared whisker input beyond baseline [24] to achieve an increase in signal to noise ratio [62].

### (e) Conclusion

We have described three different cortical homeostatic mechanisms in this study. The first is a synaptic scaling mechanism that shows a similar time-course for evoked responses in layer 2/3 and L5RS cells in the barrel cortex, and generalizes well to what is observed in layer 2/3 of the visual cortex. In layer 2/3 of the visual cortex and L5RS cells of the somatosensory cortex this mechanism is known to be TNFα-dependent. The second is a TNFα and CaMKII phosphorylation-dependent homeostatic mechanism that shows faster kinetics for evoked responses and is present in in L5IB cells. The third is a firing rate homeostasis for spontaneous activity, which is present in L5IB cells but not L5RS cells. We have not so far identified a mechanism for this form of plasticity, but observe that it can vary independently of the homeostasis of the evoked sensory responses. In the case of L5RS cells, the lack of spontaneous firing rate homeostasis is an advantage in that it increases the signal to noise ratio of the sensory response.
